# Whole-genome sequencing and comparative genomic analysis of Irpex lacteus isolated from the female reproductive tract

**DOI:** 10.1099/mgen.0.001416

**Published:** 2025-06-03

**Authors:** Yixuan Wang, Shujuan Zhang, Shi-Ling Han, Xiaomeng Ge, Shenghan Gao, Qianhui Zhu, Yadong Liu, Songnian Hu, Ziwen Jiang, Yinmei Dai, Lei Cai, Yu Vincent Fu

**Affiliations:** 1 Shandong First Medical University & Shandong Academy of Medical Sciences, Jinan, Shandong 250117, PR China; 2 State Key Laboratory of Microbial Diversity and Innovative Utilization, Institute of Microbiology, Chinese Academy of Sciences, Beijing 100101, PR China; 3 School of Chinese Materia Medica, Shenyang Pharmaceutical University, Shenyang, Liaoning 110016, PR China; 4 University of Chinese Academy of Sciences, Beijing 100049, PR China; 5 Microbial Resources and Big Data Center, Institute of Microbiology, Chinese Academy of Sciences, Beijing 100101, PR China; 6 Department of Gynecology, Beijing Obstetrics and Gynecology Hospital, Capital Medical University, Beijing Maternal and Child Health Care Hospital, Beijing 100026, PR China; 7 China General Microbiological Culture Collection Center, Institute of Microbiology, Chinese Academy of Sciences, Beijing 100101, PR China

**Keywords:** comparative genomic analysis, functional annotation, *Irpex lacteus*, phylogenetic analysis, whole-genome sequencing

## Abstract

*Irpex lacteus* is a fungus typically found on angiosperm branches and trunks. Here, we report the isolation of a novel strain of *I. lacteus* from the cervix uteri of a female patient diagnosed with endometriosis and COVID-19 at Beijing Obstetrics and Gynecology Hospital, Capital Medical University. Using whole-genome sequencing, we generated a high-quality draft genome for this human-derived strain. Comparative genomic analysis revealed significant variations amongst *I. lacteus* strains colonizing distinct environments. Functional annotations indicated that environmental adaptation has influenced specific functional traits in the strains. Moreover, potential virulence factors were identified, implying a risk of opportunistic infections associated with *I. lacteus*. This study elucidates the adaptive characteristics of *I. lacteus* across ecological niches and provides genomic insights into its opportunistic colonization.

**Impact Statement**

*Irpex lacteus* is a fungus commonly found on angiosperm branches. However, recent reports suggest its potential as an emerging human pathogen, particularly in immunocompromised individuals. In this study, we report the isolation of a novel strain, BJFC24, from the cervix of a female patient diagnosed with endometriosis and COVID-19. This discovery raises parallels with the emergence of *Cryptococcus gattii* as a human pathogen from environmental origins. Using DNBSEQ and PacBio sequencing, we assembled a high-quality genome of *I. lacteus* BJFC24, uncovering significant genomic variations between human-associated and environmental strains. Functional annotation revealed that adaptation to human environments may have shaped its pathogenic potential. Furthermore, we predicted candidate virulence factors, implying *I. lacteus* BJFC24 as a risk for human infections. This study enhances our understanding of environmental fungi to adapt to human hosts through genomic evolution, offering insights into their evolving colonization in the context of climate change and human activity.

## Data Summary

The genome of *Irpex lacteus* BJFC24 is deposited in the National Microbiology Data Center (NMDC) with accession number NMDC60197952 (https://nmdc.cn/resource/genomics/genome/detail/NMDC60197952). BioSample data are deposited in NMDC with accession number NMDC20296904 (https://nmdc.cn/resource/genomics/sample/detail/NMDC20296904). BioProject data are deposited in NMDC with accession number NMDC10019392 (https://nmdc.cn/resource/genomics/project/detail/NMDC10019392).

The phylogenetic alignments, SNP datasets and variant calling files generated in this study are available in the NMDC under accession number NMDCX0002095. The data can be accessed at https://nmdc.cn/resource/attachment/detail/NMDCX0002095.

Sequence data generated in this study are available in the National Center for Biotechnology Information Sequence Read Archive under BioProject PRJNA1247606 and BioSample accession SAMN47824703.

## Introduction

*Irpex lacteus* is a widely distributed saprophytic fungus belonging to the phylum *Basidiomycota*, subphylum *Agaricomycotina*, class *Agaricomycetes*, order *Polyporales*, family *Irpicaceae* and genus *Irpex*. It is commonly found colonizing wood in natural environments [1,3]. As the type species of the genus *Irpex* [4], *I. lacteus* was first described by Fries in 1828. This species is typically isolated from hardwood and is known to cause white rot in both living and dead tree trunks as well as fallen branches [3].

Studies have recognized *I. lacteus* as a model basidiomycete for lignin degradation [1,5, 6]. The first draft genome sequence of *I. lacteus* was reported in 2017 from a strain (F17) isolated from hardwood in Hefei City, China. This strain secretes abundant manganese peroxidase (MnP) and lignin-degrading enzymes, enabling it to degrade lignin and various aromatic pollutants [7]. Subsequent research further revealed that *I. lacteus* possesses genes encoding dye-decolourizing peroxidases, which exhibit strong oxidative capacity and stability under acidic conditions, outperforming MnPs in degrading various chemical dyes [5]. Qin *et al*. [8] showed that *I. lacteus*, cultivated on corn stalks, produces a rich arsenal of lignin, cellulose and hemicellulose-degrading enzymes. Its selective lignocellulose degradation strategy renders it a promising white-rot fungus for efficient biofuel production from straw. Furthermore, transcriptomic studies across 50 species of *Polyporales*, including *I. lacteus* CCBAS Fr 238 617/93, revealed conserved white-rot decay mechanisms on diverse lignocellulosic substrates. This decay ability is strongly linked to highly expressed lignocellulose-mobilizing oxidases, such as AA9 LPMOs, especially in association with carbohydrate-binding module (CBM) 1, which significantly boosts cellulose degradation efficiency [6].

Beyond its environmental roles, *I. lacteus* has attracted significant attention for its potential medical applications. In one study, it was isolated from *Cordyceps hawkesii* Gray and identified as a source of cordycepin, thereby expanding its pharmaceutical potential [9]. Moreover, polysaccharides derived from a mutant strain (ILN3A) have demonstrated effectiveness in ameliorating membranous glomerulonephropathy, suggesting their potential as anti-inflammatory agents for the treatment of chronic glomerulonephritis [10]. Additionally, *I. lacteus* polysaccharides have also shown potential in treating polycystic ovary syndrome, by improving metabolic disorders, regulating hormone levels, reducing oxidative stress and mitigating ovarian fibrosis [11].

In clinical settings, *I. lacteus* has occasionally been reported in immunocompromised patients. In 2005, it was identified as a possible causative agent of pulmonary abscess in an immunosuppressed child through morphological characteristics and molecular phylogenetic analysis [12]. A subsequent case identified *I. lacteus* in the cerebrospinal fluid of an elderly male with sarcoidosis, where elevated serum *β*-d-glucan and galactomannan levels correlated with *I. lacteus* infection [13]. Recently, *I. lacteus* was isolated from bronchoscopy specimens in a lung transplant recipient, where initial colonization was asymptomatic, suggesting a relatively low virulence [14]. These findings highlight the dual nature of *I. lacteus*: environmental strains contribute to the decomposition of dead wood and play an important role in lignin degradation and nutrient cycling; meanwhile, as a potential opportunistic pathogen, it underscores the necessity of analysing the functional characteristics of human-derived strains.

In this study, a fungal strain (BJFC24) was isolated from the cervix uteri of a woman diagnosed with endometriosis complicated by coronavirus disease 2019 (COVID-19). Employing multi-locus phylogeny and morphological characters, this strain was identified as *I. lacteus*. To investigate the genomic basis of this newly isolated strain, whole-genome sequencing was performed using DNBSEQ and PacBio sequencing. The high-quality genome sequence of *I. lacteus* BJFC24 enabled comparative genomics with other strains from diverse environmental sources. The average nucleotide identity (ANI), genome structure variations and the embodied biological characteristics of these strains across distinct ecological niches were analysed, which provided valuable insights into their taxonomy and opportunistic colonization.

## Methods

### Strain isolation

The strain BJFC24 was isolated from a cervical swab sample collected from a patient diagnosed with endometriosis complicated with COVID-19 at Beijing Maternity Hospital of Capital Medical University, Chaoyang District, Beijing, China. The sample was collected using a sterile cotton swab, immediately placed in sterile saline solution and transported to the laboratory within 2 h for cultivation. A 100 µl aliquot of the sample was inoculated onto potato dextrose agar (PDA) medium (3.0 g l^−1^ potato extract, 20.0 g l^−1^ glucose and 20.0 g l^−1^ agar powder) supplemented with 500 µl l^−1^ chloramphenicol (50 mg ml^−1^) and incubated at 37 °C under aerobic and dark conditions. The strain was subcultured on PDA at least twice to obtain pure cultures.

### Morphological observation

Pure cultures grown on PDA medium were prepared for microscopic examination. Micromorphological characteristics, including pigmentation and size of hyphae in 10% potassium hydroxide (KOH), cotton blue (CB) and Melzer’s reagent (IKI). Morphological characteristics were examined and photo-documented under a Nikon 80i compound microscope with differential interference contrast optics. Scanning electron microscopy (SEM) was conducted at the Beijing Regional Center of Life Science Instruments, Chinese Academy of Sciences. *I. lacteus* BJFC24 was incubated on a PDA medium at 37 °C for 8 days, then soaked in glutaraldehyde at 4 °C for 23 h. The fixed samples were dehydrated through a graded ethanol series (70%, 85%, 95% and 100%). Following dehydration, the samples were critical-point dried with liquid CO_2_ using a Leica EM CPD300 and then sputter-coated with gold-palladium (Hitachi E-1045 ion sputter). The specimens were observed and photographed using a Field Emission Scanning Electron Microscope (SU8010, Hitachi).

### DNA extraction, amplification and phylogenetic analysis based on internal transcribed spacer and LSU sequences

DNA was extracted from fresh mycelium grown on PDA using the HP Fungal DNA Extraction Kit (Omega Bio-tek). PCR amplification was carried out in a total volume of 50 µl, containing 25 µl of 2×Super PCR Mix (with dye, Liuhe Huada Gene Technology Co., Ltd., Beijing, China), 1 µl each of 10 pmol µl^−1^ forward and reverse primers and 3 µl of genomic DNA, adjusted with distilled deionized water. The internal transcribed spacer (ITS) region of the rDNA and the 28S LSU of the rDNA regions were amplified and sequenced using primers ITS1/ITS4 [15] and LR0R/LR5 [16], respectively.

Sequences from each single-gene dataset were aligned using the default settings of MAFFT v7.505 [17], and the resulting alignments were imported into mega v7 [18] to trim non-conserved terminal regions and manually fine-tune the alignments for sequence consistency. Phylogenetic analysis was performed based on a combined ITS and LSU dataset using maximum likelihood (ML) and Bayesian inference (BI) methods through the CIPRES Science Gateway portal (https://www.phylo.org/) [19]. The ML analysis was conducted with RAxML-HPC2 v8.2.12 [20] under the GTR+GAMMA model, generating the best-scoring tree from 1,000 bootstrap replicates. For BI analysis, MrModelTest (v2.4) [21] was used to determine the optimal evolutionary model for each gene region in all datasets based on the Akaike information criterion. A Markov Chain Monte Carlo chain was then run for 10 million generations, with sampling every 1,000 generations. During the ‘burn-in period’, the first quarter of the generated trees was discarded, whilst the remaining trees were used to construct a 50% majority-rule consensus phylogenetic tree with posterior probabilities (PP). RAxML support values (ML-BS≥70%) and Bayesian PP (BI-PP≥0.90) were annotated on the tree. The tree was visualized using FigTree v1.4.2 (http://tree.bio.ed.ac.uk/software/figtree). Alignment details for the sequences used in this study are provided in Table S1, available in the online version of this article.

### Contamination controls

To ensure the reliability of strain isolation and genomic analyses, stringent contamination control measures were implemented throughout the experimental process. The hospital sampling room was disinfected using UV light prior to sample collection. During the procedure, the patient’s sampling area was prepared using a vaginal speculum that had been sterilized by high-pressure steam, and sterile swabs were used for cervical sampling. Immediately after collection, the swab was immersed in sterilized saline solution and then placed in a sealed container for vertical transport to the laboratory for subsequent cultivation.

In the laboratory, all sample inoculation and strain subculturing operations were performed in a laminar flow hood, near an alcohol lamp, to maintain optimal aseptic conditions. A blank control was set during the sample collection, which was identical to the cervical sample collection, except that the sterile swab was not inserted into the vaginal speculum to swab the cervix. The blank control showed no contamination.

During strain DNA extraction, negative controls were included. Each negative control consisted of all the extraction reagents but no biological sample. These controls were processed concurrently with the test samples and subjected to PCR amplification. The absence of detectable amplification in the negative controls confirmed that the DNA extraction process was free from contamination.

### Genomic DNA extraction, sequencing and assembly

For extracting genomic DNA, a small amount of mycelium was picked from a PDA agar plate and inoculated into a test tube containing 10 ml of potato dextrose broth medium. The culture was incubated at 37 °C with shaking at 200 r.p.m. After removing the liquid medium, the mycelium was ground with liquid nitrogen, and genomic DNA was extracted using the HP Fungal DNA Extraction Kit (Omega Bio-tek Company).

For sequencing, we adopted a combination of second- and third-generation sequencing technologies to ensure high-quality genome assembly with enhanced completeness and accuracy. The PacBio Revio platform (BGI Shenzhen) was employed. Genomic DNA samples were sheared into 20–40K fragments using g-TUBE tubes, followed by enzyme digestion, damage repair, end repair and ligation of dumbbell-shaped adapters to form an SMRTbell hairpin structure. The BluePippin was used for library size selection. Once the library passed quality control, it was sequenced on the Pacbio Revio platform.

For the next-generation sequencing, the DNBSEQ platform was utilized. Genomic DNA was fragmented, selected and prepared with end repair, A-tailing at the 3′ end and adapter ligation. Following PCR amplification and quality control, the PCR products were denatured into single strands, and circularization was performed to obtain single-stranded circular DNA. Un-circularized DNA was digested, and single-stranded circular DNA molecules were amplified by rolling circle amplification to form DNA nanoballs (DNBs) containing multiple copies. These DNBs were added to high-density chips for joint probe-anchored synthesis sequencing.

SOAPnuke v1.5.6 [22] was used to filter the raw sequencing data to remove low-quality reads, adapters and duplication contamination. The genome was assembled using hifiasm v0.19.9 [23], with format conversion performed using gfatools v0.4 (https://github.com/lh3/gfatools). NextPolish2 (v0.2.0) was used for genome correction to obtain the final assembly [24]. Genome assembly quality was assessed using QUAST v5.2.0 [25], and completeness was evaluated using BUSCO v5.7.1 [26] with the fungi_odb10 reference database. The genome file was statistically analysed using SeqKit v2.5.0 [27].

### Genome function prediction and annotation

Using the basic workflow outlined on Funannotate’s website (https://funannotate.readthedocs.io/en/latest/; accessed on 29 May 2024), genome prediction and annotation were performed with Funannotate v1.8.15 [28]. This process included cleaning, sorting, masking of repetitive sequences and gene prediction, resulting in a non-redundant and complete gene set. Functional annotation of genes was then carried out with Funannotate using the default parameters.

To further predict the functional potential of the genome, we performed annotations from multiple perspectives. Gene Ontology (GO) terms [29] and EuKaryotic Orthologous Groups (KOG) [30] were annotated using eggNOG-mapper v2.1.12 [31] with eggNOG orthology data v5.0.2 [32]. Kyoto Encyclopedia of Genes and Genomes (KEGG) pathway annotation was conducted using the KAAS software [33] with online blast annotation (parameters set for bi-directional best hit and for eukaryotes). Carbohydrate-active enzymes (CAZymes) [34] were annotated via HMMer search against dbCAN v12.0 [34,35].

Secondary metabolite gene clusters were predicted using antiSMASH (v6.1.1)[36]. Pathogenicity-related genes were screened against the Pathogen-Host Interaction Database (PHI v4.17 http://www.phi-base.org/, accessed on 3 July 2024) [37] and the Database of Fungal Virulence Factors (DFVF, http://sysbio.unl.edu/DFVF/, accessed on 3 July 2024) [38], with all blast comparisons performed under conditions where E≤1E-05.

### Reference genome collection and ANI analysis

We collected genomes from the NCBI database corresponding to the species name *I. lacteus* used in this study as reference sequences, including *I. lacteus* F17 [7], *I. lacteus* CCBAS Fr 238 617/93 [6] and *I. lacteus* Y8604A (https://www.ncbi.nlm.nih.gov/nuccore/MQVO00000000.2, JABYQL000000000.1 and JJAXQKJ000000000.1, accessed on 29 May 2024). For *I. lacteus* Y8604A, only sequences longer than 1,000 bp were retained. These reference genomes were processed using the same workflow as described for *I. lacteus* BJFC24 and were subsequently included in comparative analyses. ANI is a measure of genetic continuity between microbial genomes at the nucleotide level. To analyse the similarity between the genomes of different strains, we used FastANI v1.33 [39] to calculate the ANI of *I. lacteus* genomes.

### Genomic sequence variation calling

Clean reads from the strain isolated in this study were aligned to the reference genomes of *I. lacteus* F17, *I. lacteus* CCBAS Fr 238 617/93 and *I. lacteus* Y8604A using BWA-MEM v0.7.18 [40]. The resulting alignment files were subjected to format conversion and indexing with samtools v1.20 [41]. Variant calling, including the identification of SNPs and insertions/deletions (InDels), was performed using GATK v4.5.0.0 [42]. This process involved reordering alignments, marking duplicate reads and filtering out low-quality variants. The filtering criteria were applied using GATK VariantFiltration with the following parameters: ‘QD <2.0 || FS >60.0 || MQ <40.0 || MQRankSum <−12.5 || ReadPosRankSum <−8.0 || SOR>3.0 || DP <10’. High-quality SNPs and InDels were extracted using GATK SelectVariants. Further refinement of the variant callings was achieved using Vcftools v0.1.16 [43] to generate the final VCF file. Annotation of variant sites was carried out with snpeff v5.2 [44] based on the reference genome.

## Results

### Phylogenetic analyses and morphological observation of *I. lacteus* BJFC24

From the cervix uteri swab, we obtained white mycelium on the PDA plate supplemented with chloramphenicol. To identify this strain, the ITS and LSU regions were amplified using PCR and subsequently sequenced.

**Fig. 1.**
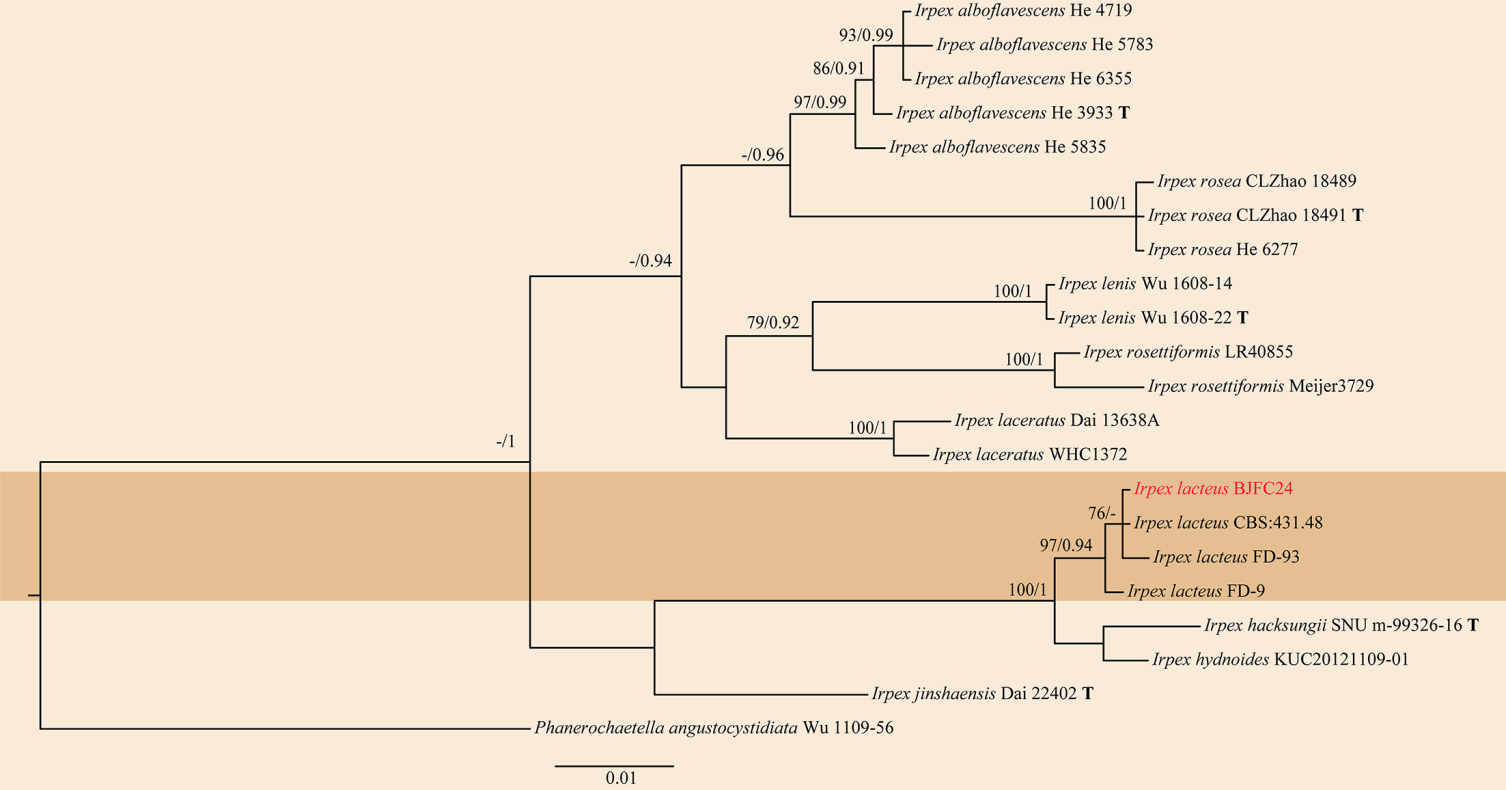
The phylogenetic tree was inferred based on the combined ITS and LSU gene regions of *Irpex* species, using *Phanerochaetella angustocystidiata* Wu 1109-56 as the outgroup. The strains isolated in this study are indicated in red. The RAxML bootstrap support values (ML-BS≥70%) and Bayesian PP (BI-PP≥0.90) are displayed; type strains (ex-type) are indicated in bold with T.

The multi-locus alignment covered 1,113 bases (including gaps) across both gene regions. The optimal nucleotide substitution model for the ITS was HKY+I+G, whereas GTR+I was chosen for the LSU. The topology of multi-locus phylogenetic trees obtained from ML and BI analyses was identical. These results indicated that the strain BJFC24 examined in this study clustered with the representative strain CBS:431.48 of *I. lacteus* (Figs 1, S1 and S2). Furthermore, the ITS and LSU sequences of these two strains, CBS:431.48 and BJFC24, share high similarity (99.88%). Thus, the strain was identified as *I. lacteus*.

**Fig. 2.**
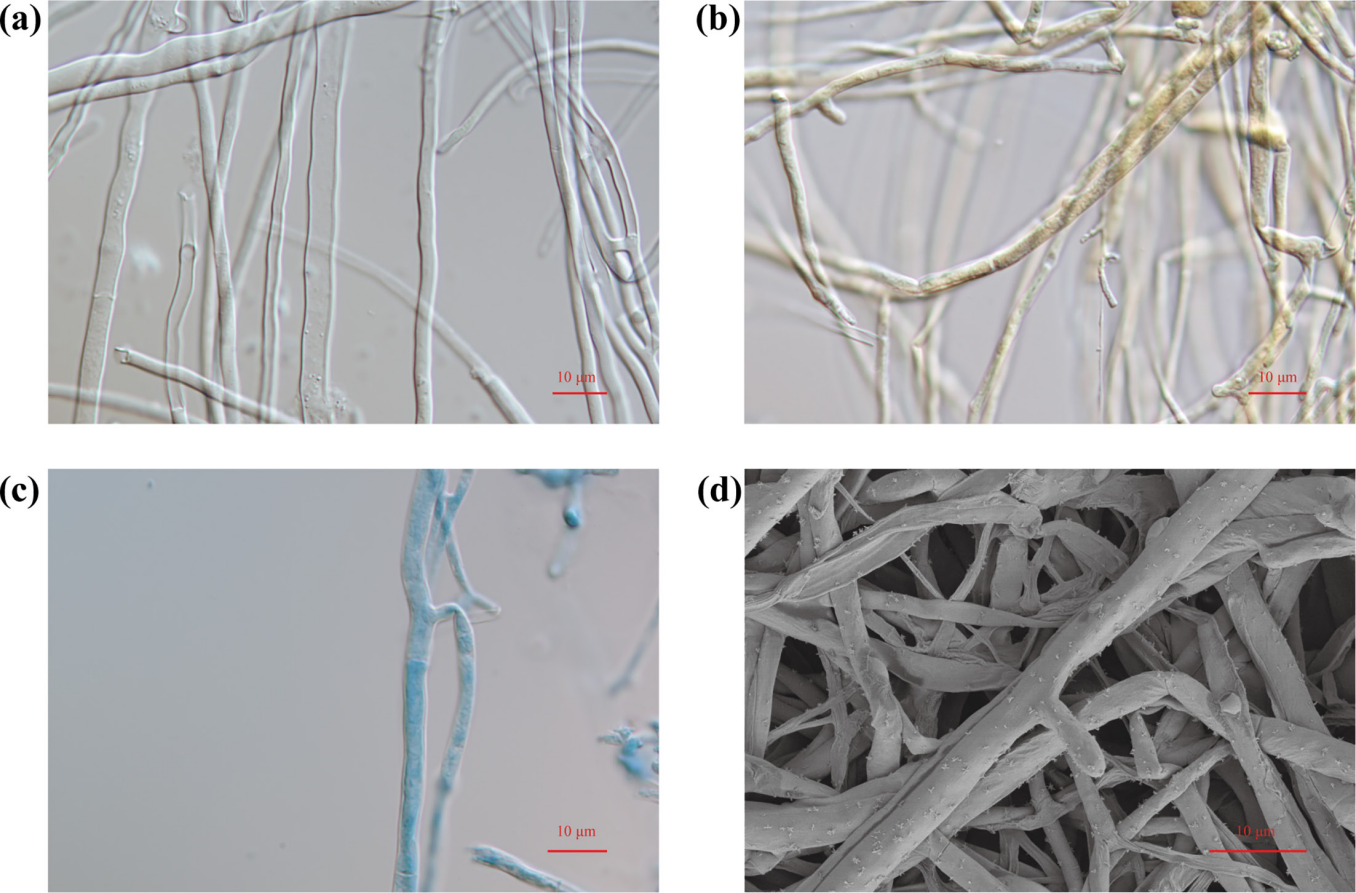
Morphological characterization of *Irpex lacteus* BJFC24 mycelia observed under an optical microscope at 100× magnification. (**a**) Mycelia visualized using the KOH wet mount method. Mycelia were stained with (**b**) IKI or (**c**) CB. (**d**) SEM image of the fungal cells.

The BJFC24 strain was cultured on PDA medium at 37 °C for 8 days, forming a layer of white mycelium covering the Petri dish. Under an optical microscope at 100× magnification, the mycelial structure (Fig. 2a) showed transparent, septate, branching hyphae with diameters ranging from 2.05 to 5.12 µm. Melzer’s reagent staining yielded a pale yellow-brown coloration of the hyphae (Fig. 2b), indicating a negative reaction, whilst a positive CB stain indicated the presence of cyanophilic hyphae (Fig. 2c). SEM revealed separated, bifurcated hyphae, as illustrated in Fig. 2(d).

In order to obtain further evidence supporting the colonization of *I. lacteus* BJFC24 at the human cervix uteri, we cultured the cells under conditions mimicking the cervical environment. Although the cervical environment is dynamic, it is generally characterized by a temperature of 37 °C and a pH range of 5.4–8.2 [45]. *I. lacteus* BJFC24 exhibited robust growth at 37 °C and pH 6.0.

### Basic characteristics of the *I. lacteus* genomes

As this is the first report of *I. lacteus* inhabiting the female genital tract, we sought to explore the underlying mechanisms that enable *I. lacteus* BJFC24 to adapt to the human reproductive tract environment. To this end, we performed whole-genome sequencing of *I. lacteus* BJFC24. The assembly comprises a total of 79 contigs, with a total length of 44,332,066 bp, a G+C content of 48.39 mol% and an N50 of 3,517,405 bp, achieving a genome completeness of 99.4%. Detailed information on the four *I. lacteus* strains, *I. lacteus* BJFC24, *I. lacteus* F17, *I. lacteus* CCBAS Fr 238 617/93 and *I. lacteus* Y8604A, is provided in Table 1, and their genomic characteristics are summarized in Table 2. The assembly yielded a higher N50 and average sequence length compared to the other three strains, suggesting that the genomic assembly maintains high quality and integrity.

**Table 1.** Summary of basic information on strains used in this study

Strain	F17	CCBAS Fr 238 617/93	Y8604A	BJFC24
Name	*I. lacteus*	*I. lacteus*	*I. lacteus*	*I. lacteus*
Isolation source	Decayed wood chips	Woods [79]	*Homo sapiens*, faeces	*Homo sapiens*, cervical swab
Origin	Hefei, China	USA	Dalian, China	Beijing, China
Sequencing technology	PacBio	PacBio	Illumina HiSeq	PacBio
Assembly method	PacBio SMAT v2.3.0 [7]	Falcon v0.7.3 [6]	SPAdes v3.15.5 (https://www.ncbi.nlm.nih.gov/nuccore/JJAXQKJ000000000.1, accessed on 29 May 2024)	hifiasm v0.19.9
Accession number	MQVO00000000	JABYQL000000000	JAXQKJ000000000	NMDC60197952
Original study	Genome sequence of the white-rot fungus *Irpex lacteus* F17, a type strain of lignin degrader fungus [7]	Gene family expansions and transcriptome signatures uncover fungal adaptations to wood decay [6]	Unpublished lab collection	This study

**Table 2.** Genome assembly features of *I. lacteus*

Statistics	*I. lacteus* F17	*I. lacteus* CCBAS Fr 238 617/93	*I. lacteus* Y8604A	*I. lacteus* BJFC24
Number of contigs	317	424	2472	79
Contigs (>=50,000 bp)	81	151	222	60
Total length (bp)	44,362,654	47,742,868	37,821,090	44,332,066
Max contig length (bp)	3,253,204	2,963,555	444,834	4,080,045
Average sequence length (bp)	139,945.3	112,601.1	15,299.8	561,165.4
N50 (bp)	1,154,715	476,280	71,622	3,517,405
G+C (mol%)	49.47	50.67	50.92	48.39
BUSCO completeness of genome (%)	89.6	97.7	98.1	99.4

### Gene prediction and functional annotation of *I. lacteus* BJFC24

#### Annotation of genes related to basic function

A total of 11,741 CDSs were predicted in the *I. lacteus* BJFC24 genome (Table 3) and functionally annotated across KOG, GO, KEGG and CAZy databases (Table 3). These analyses aim to uncover the genetic basis underlying *I. lacteus* BJFC24’s environmental adaptability and functional potential.

**Table 3.** Genomic component analysis

	*I. lacteus* F17	*I. lacteus* CCBAS Fr 238 617/93	*I. lacteus* Y8604A	*I. lacteus* BJFC24
BUSCO completeness of protein (%)	85.1	88.5	94.1	96.7
Total genes	12,764	15,061	12,070	12,454
Protein-coding genes	12,248	14,875	11,921	11,741
With a KOG number	8120	9907	7895	7767
With a GO number	3656	4541	3553	3542
With a KEGG number	2485	2920	2429	2471
With a CAZy number	366	421	353	358
Secondary metabolite biosynthetic gene clusters	26	33	26	24
With a PHI number	2684	3290	2603	2602
With a DFVF number	1085	1288	1048	1053

In the KOG analysis, 7,767 genes were categorized into 24 classifications (Fig. S3 and Table 3). Significant numbers of genes were associated with post-translational modification; protein turnover; chaperones (O); signal transduction mechanisms (T); carbohydrate transport and metabolism (G); secondary metabolite biosynthesis; transport and catabolism (Q); energy production and conversion (C); transcription (K); intracellular trafficking, secretion and vesicular transport (U); translation, ribosomal structure and biogenesis (J); and amino acid transport and metabolism (E). This indicates that *I. lacteus* BJFC24 has extensive potential for protein modification and intracellular trafficking.

In the GO analysis, 3,542 genes were assigned GO terms (Table 3). Within the molecular function ontology, genes were primarily associated with catalytic activity, binding, ATP-dependent activity and transporter activity. In the ontology of cellular components, most genes were predicted to be cellular anatomical entities and protein-containing complexes. In biological processes, the highest gene counts were related to cellular and metabolic processes (Fig. S4A). These findings underscore the importance of cellular functions such as metabolism and protein activity, suggesting that *I. lacteus* BJFC24 possesses the molecular machinery required for fundamental biological processes.

#### Annotation of genes related to metabolites

For KEGG analysis, 2,471 genes were annotated to the KEGG pathways. The pathways related to microbial metabolism and survival were selected for further study (Table 3 and Fig. S4B). Most genes were mapped to pathways involved in signal transduction, transport and catabolism, carbohydrate metabolism and amino acid metabolism, consistent with KOG functional predictions. Additionally, translation, transcription and immune system functions were similarly annotated in both KOG and GO annotations. This suggests a broad metabolic capacity and functional versatility of *I. lacteus* BJFC24.

Based on genome annotation and dbCAN analysis, the genome of *I. lacteus* BJFC24 contains a total of 193 genes encoding glycoside hydrolases (GHs), which is the highest family within the identified CAZyme repertoire. It was followed by 84 genes encoding auxiliary activity enzymes (AAs), 6 encoding CBMs, 14 encoding carbohydrate esterases, 56 encoding glycosyltransferases and 11 genes encoding polysaccharide lyases (Fig. S5A). Given that *I. lacteus* naturally possesses the capacity for lignocellulose degradation, the CAZyme prediction profile in this study also highlights a significant presence of genes in the AA and GH families, which are essential for cellulase, hemicellulase and oxidoreductase activities. This observation aligns with previous studies [6,8], indicating that although BJFC24 has the ability to colonize the human body, it retains lignocellulose-degrading characteristics linked to plant-associated traits.

Fungi are renowned for producing secondary metabolites with diverse biological activities, including antimicrobial, immunosuppressive and toxic effects [36,46, 47]. These secondary metabolites may facilitate fungal adaptation to specific ecological niches. Using the antiSMASH platform, we screened the BJFC24 genome and identified 24 gene clusters linked to secondary metabolism, categorized as type I polyketide synthase (T1PKS), type III polyketide synthase, non-ribosomal peptide synthetase-like (NRPS-like), NRPS-like-T1PKS, betalactone and terpene (Table 3 and Fig. S5B). Amongst them, NRPS-like clusters were the most abundant, whereas betalactone and T1PKS clusters contained relatively fewer predicted genes. It implies that during colonization of the female reproductive tract, BJFC24 might rely on an enhanced number of gene clusters associated with non-ribosomal peptide production to better adapt to this environment.

#### Prediction of pathogenic genes in *I. lacteus* BJFC24

To explore the potential interactions between *I. lacteus* BJFC24 and its host, we performed predictive analyses using the PHI database. Diamond blastp analysis identified 2,602 genes in *I. lacteus* BJFC24 linked to pathogen-host interactions that fell into eight phenotypic categories (Fig. S5C). Whilst the majority of these genes (1,510 genes) were associated with reduced virulence, 156 genes were linked to increased virulence (hypervirulence). Despite the overall trend towards attenuated virulence, the presence of hypervirulence-associated genes implies that *I. lacteus* BJFC24 may pose an opportunistic pathogenic risk under certain conditions, particularly in immunocompromised hosts.

Given the limited scope of the PHI database [38], we further utilized the DFVF database to gain additional insights into the virulence factors of *I. lacteus* BJFC24. The DFVF analysis identified 569 out of 1,053 virulence factor genes that are linked to human diseases (Table S2), further indicating its potential pathogenicity. These genes were implicated in diseases such as invasive candidal disease (285 genes), cryptococcosis (89 genes), infection (65 genes) and occasional infection (50 genes). Notably, these 569 virulence factor genes were found to be orthologous to those in 26 pathogenic fungi (Table S2), with highlighting the 17 species showing the highest homology to *I. lacteus* BJFC24 (Table 4 and Fig. S5D). This homology may explain *I. lacteus* BJFC24’s ability to colonize the reproductive tract and potentially exert opportunistic pathogenic effects.

**Table 4.** Homology-based species predictions for *I. lacteus* BJFC24 derived from the DFVF database

Species	Host	Disease	Percent (%)
*Candida albicans*	Humans	Invasive candidal disease	49.91
*Cryptococcus neoformans*	Humans	Cryptococcosis	14.94
*Neosartorya fumigata*	Humans	Infection	7.73
*Saccharomyces cerevisiae*	Humans	Occasional infection	3.34
*Paracoccidioides brasiliensis*	Humans	Paracoccidioidomycosis	2.99
*Ajellomyces dermatitidis*	Humans	Cutaneous infection	2.81
*Pichia pastoris*	Humans	Occasional infection	2.81
*Ajellomyces capsulata*	Humans	Darling’s disease	2.64
*Arthroderma otae*	Humans	Dermatophytoses	2.46
*Trichophyton verrucosum*	Humans	Infection	1.41
*Hypocrea virens*	Humans	Infection	1.23
*Scheffersomyces stipitis*	Humans	Occasional infection	1.23
*Coccidioides posadasii*	Humans	Coccidiomycosis	1.05
*Verticillium fungicola*	Humans	Verticillium disease	1.05
*Candida glabrata*	Humans	Occasional infection	0.70
*Cryptococcus gattii*	Humans	Cryptococcosis	0.70
Others	–	–	2.99

### Genome evolutionary and comparative analysis of different *I. lacteus* strains

#### Genomic similarity analysis

To investigate the genomic similarity between human-derived and environmental strains of *I. lacteus*, we conducted an ANI analysis across multiple strains. The ANI values amongst four strains of *I. lacteus* were ~94% (Fig. S6). The results show that *I. lacteus* BJFC24 did not exhibit significantly higher similarity to any of the other three strains. These findings suggest potential genetic differentiation that may be relevant to their adaptation to different environments or hosts and prompted us to investigate the genomic variations that may enable environmental strains to adapt to human-associated niches.

#### Genomic variation analysis

To further explore genomic differences and evolutionary relationships, the clean reads of isolate BJFC24 were mapped to the reference genome of isolates F17, CCBAS Fr 238 617/93 and Y8604A. The analysis identified SNPs and InDels, which are shown in Table 5. The gut-derived strain Y8604A exhibited the fewest SNPs when compared to BJFC24, indicating a higher genomic sequence similarity between the gut-derived strain Y8604A and cervix-uteri-derived BJFC24. In contrast, the environmental strains CCBAS Fr 238 617/93 and F17 displayed a greater number of genomic variations when compared to BJFC24 and Y8604A, suggesting that the two environmental strains have undergone more extensive evolutionary differentiation.

**Table 5.** Variant summary of *I. lacteus* BJFC24 relative to three strains within the same species

	*I. lacteus* F17	*I. lacteus* CCBAS Fr 238 617/93	*I. lacteus* Y8604A
SNPs	753,717	780,742	512,407
Ts/Tv (transitions/transversions) ratio	2.48	2.47	2.56
Insertion	90,609	110,442	97,619
Deletion	88,075	87,252	95,667
InDels	178,684	197,694	193,286

**Fig. 3.**
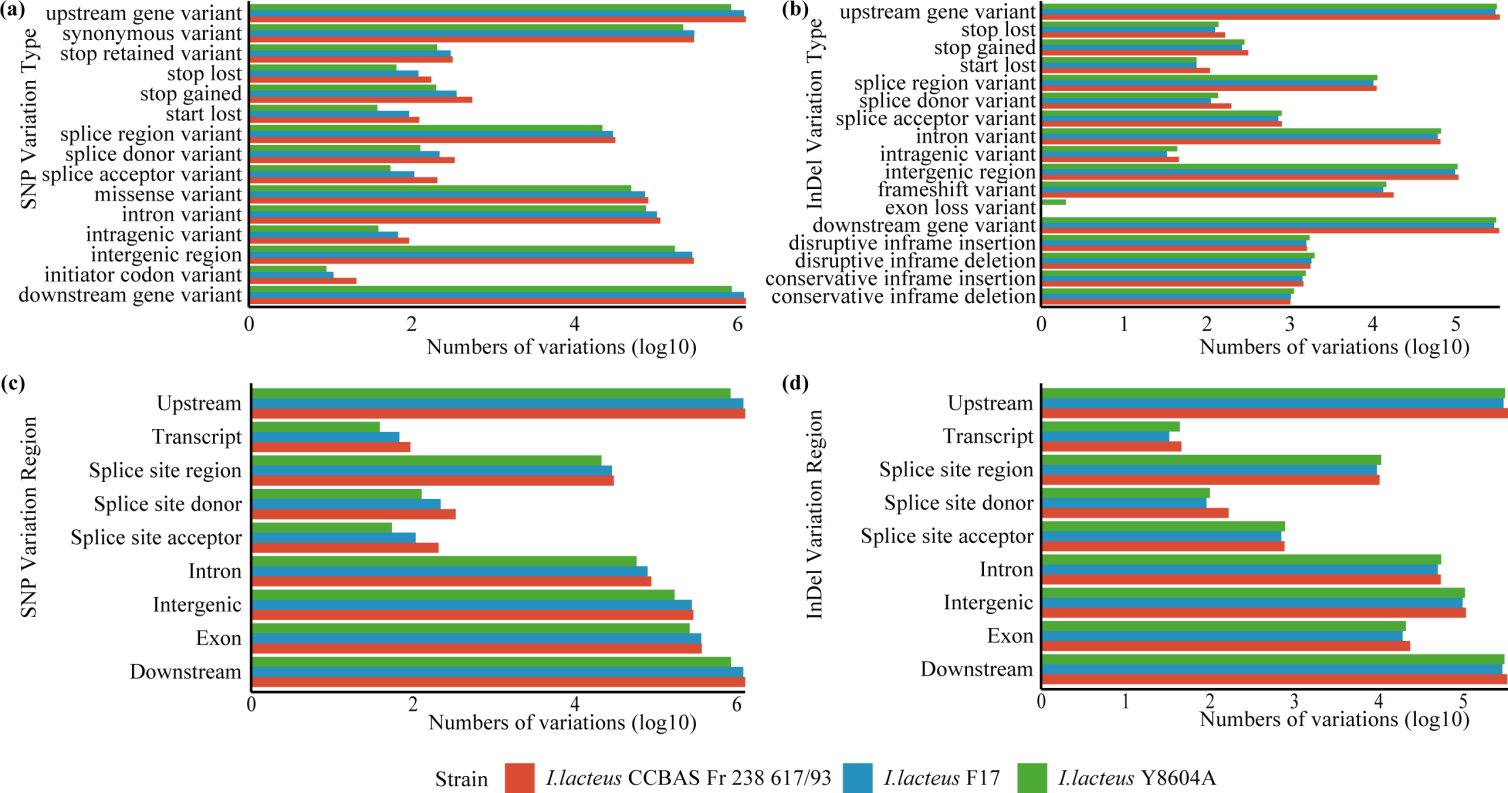
Genome types and regions of variations (identified by comparison with the reference genome of strain BJFC24) in three other *I. lacteus* isolates. The number of SNP (**a, c**) and InDel (**b, d**) variation types and regions across the genomes is presented on a log10 scale.

The transition/transversion ratio exceeded the criterion of random mutation (0.5), implying a higher frequency of transitions in strains from different ecological niches. The SNP variation results, categorized by genomic position and type, are illustrated in Fig. 3(a, c), as well as in Table S3. Most variations were located in the upstream and downstream regions (Fig. 3a), which is consistent with InDel findings, with exon, intergenic and intron regions following (Fig. 3b). Interestingly, InDel variations were more abundant in the intron region than in the exon region. Regarding the types of InDel variations (Fig. 3d), the number of frameshift variant mutations in *I. lacteus* strains Y8604A and BJFC24 was higher than in strain F17 but lower than in strain CCBAS Fr 238 617/93. These results suggest that environmental pressures influence genetic variation in colonizing strains.

#### Comparative genomic analysis

Comparative genomic analyses were conducted to explore the functional differences in the genomes of four *I. lacteus* strains (*I. lacteus* BJFC24, *I. lacteus* F17, *I. lacteus* CCBAS Fr 238 617/93 and *I. lacteus* Y8604A) and to identify strain-specific genetic features related to adaptation to distinct environments, potential virulence factors and interactions with their hosts. Annotations from KOG, KEGG, GO, CAZy, PHI, DFVF and antiSMASH databases (Table 3) were used to identify these functional differences. To ensure consistency and enable accurate cross-genome comparisons, the same annotation framework was applied uniformly across these four genomes.

##### Comparative genomic analysis of basic function genes

**Fig. 4.**
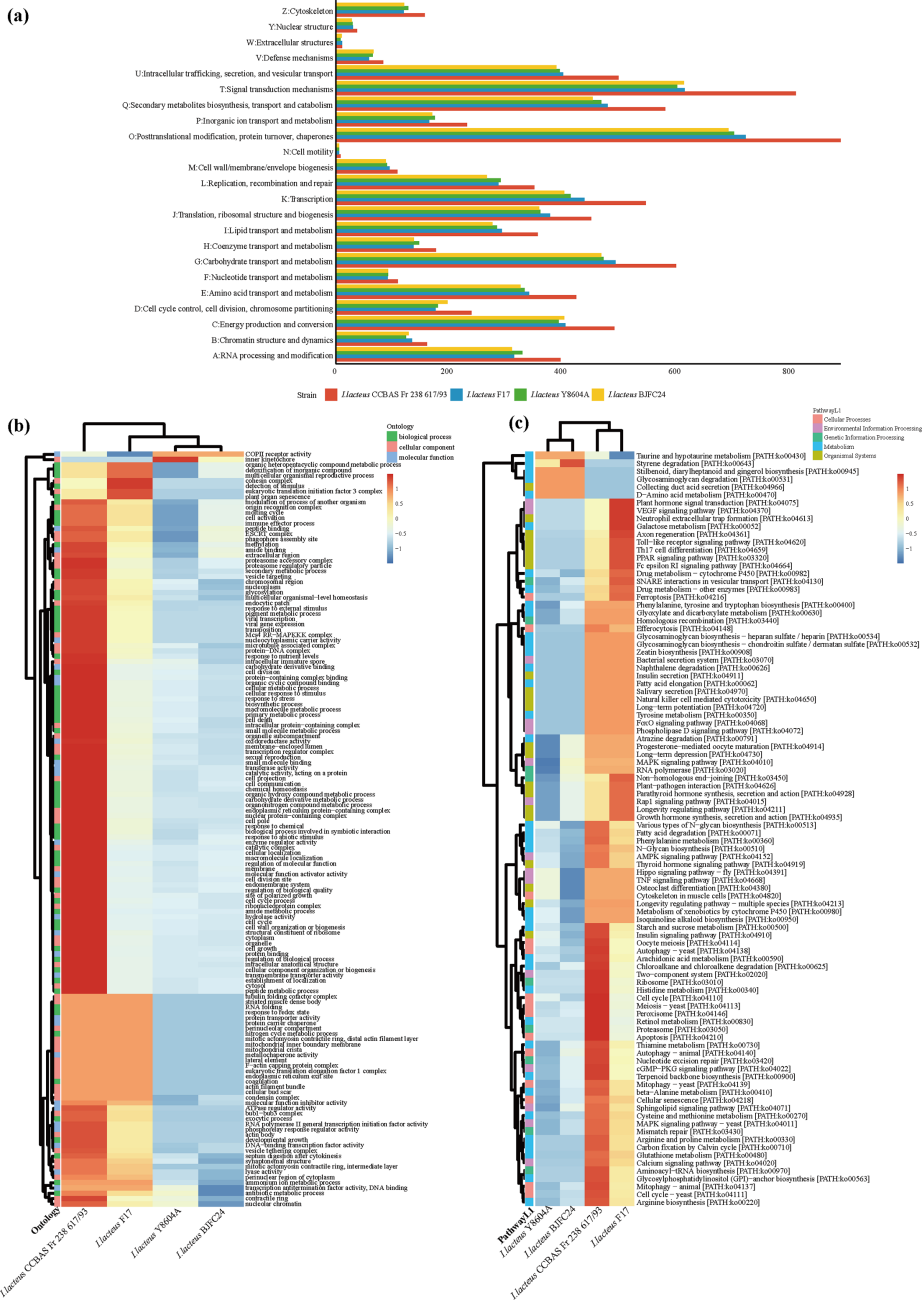
Annotation of *I. lacteus* strains. (**a**) Comparative analysis of the number of genes annotated to KOG categories between human-derived and environmental strains. (**b**) Heatmap illustrating GO term difference between human-derived and environmental strains. (**c**) Heatmap displaying KEGG pathway differences between human-derived and environmental strains.

The KOG analysis revealed that 15 functional categories contained more genes in environmental strains than in human-derived strains (Fig. 4a and Table S4). Most of these genes are associated with functions such as post-translational modification, protein turnover, chaperoning, signal transduction, carbohydrate transport and metabolism, biosynthesis and catabolism of secondary metabolites, transcription, energy production and conversion, intracellular trafficking, secretion, translation and ribosomal structure and biogenesis, as well as amino acid, lipid and cell wall metabolism. This indicates that environmental strains may possess enhanced capacities for metabolic versatility and cellular adaptation compared to human-derived strains. Notably, many of these same categories (e.g. post-translational modification, signal transduction and secondary metabolism) were amongst the represented KOG classes in BJFC24 itself (Fig. S3), underscoring that BJFC24 retains the broad functional repertoire characteristic of the species. The reduction of genes in these categories amongst human-derived strains may reflect niche-specific genomic streamlining associated with host adaptation.

In the GO analysis, human-derived strains exhibited a higher gene count associated with coat protein complex II (COPII) receptor activity and inner kinetochore, relating to cellular transport and mitotic function (Fig. 4b and Table S5). Conversely, environmental strains displayed 140 GO functional annotations with higher gene counts than human-derived strains (Fig. 4b and Table S5), particularly in categories related to cellular and macromolecule metabolic processes, biosynthesis and intracellular anatomical structure. Furthermore, these environmental strains showed a greater number of genes involved in binding small molecules, organic cyclic compounds and proteins, as well as in various enzymatic functions. This suggests an enhanced capacity for adapting to significant environmental fluctuations through intricate metabolic regulation and molecular interactions. External environmental conditions, such as nutrient scarcity or temperature fluctuations, tend to vary more drastically than those within a human host. Similarly, these comparative trends are reflected in BJFC24’s own GO annotations (Fig. S4A), which were dominated by terms related to biological processes and cellular components, and these functions were essential for both environmental survival and opportunistic host colonization. As a human-derived isolate, BJFC24 retains metabolic and transport‐related genes whilst also exhibiting features that facilitate host adaptation.

##### Comparative genomic analysis on metabolism genes

Building upon this, the KEGG analysis further focused on microbial metabolism and survival pathways, and the results revealed that environmental isolates exhibited a greater number of genes across 90 KEGG Orthology pathways, whereas human-derived isolates had more genes in only six pathways (Fig. 4c and Table S6). Environmental strains exhibited relatively higher counts of genes annotated to signal transduction, transport and catabolism, carbohydrate metabolism and amino acid metabolism. These pathways were also prevalent in BJFC24’s annotations (Fig. S4B and Table S6), indicating that BJFC24 retains genes associated with these metabolic functions. Key pathways in environmental strains included ribosome, peroxisome, cell cycle, autophagy, nucleotide excision repair and amino acid metabolism. This suggests that environmental strains require a broader functional gene diversity to adapt to complex natural surroundings, particularly in metabolism-related pathways. Pathways related to meiosis and mitogen-activated protein kinases (MAPK) signalling were also present, indicating that environmental strains actively engage in metabolic processes to support growth, replication, repair and signal transduction, granting them a competitive advantage in natural settings.

Human-derived strains, however, demonstrated a higher number of genes associated with specific metabolic pathways, including taurine and hypotaurine metabolism, styrene degradation, stilbenoid/diarylheptanoid/gingerol biosynthesis, glycosaminoglycan degradation, collecting duct acid secretion and d-amino acid metabolism. Notably, flavone and flavonol biosynthesis and peptidoglycan biosynthesis were unique to strains colonizing the reproductive tract (Table S6), underscoring the specificity of strain to its host environment.

**Fig. 5.**
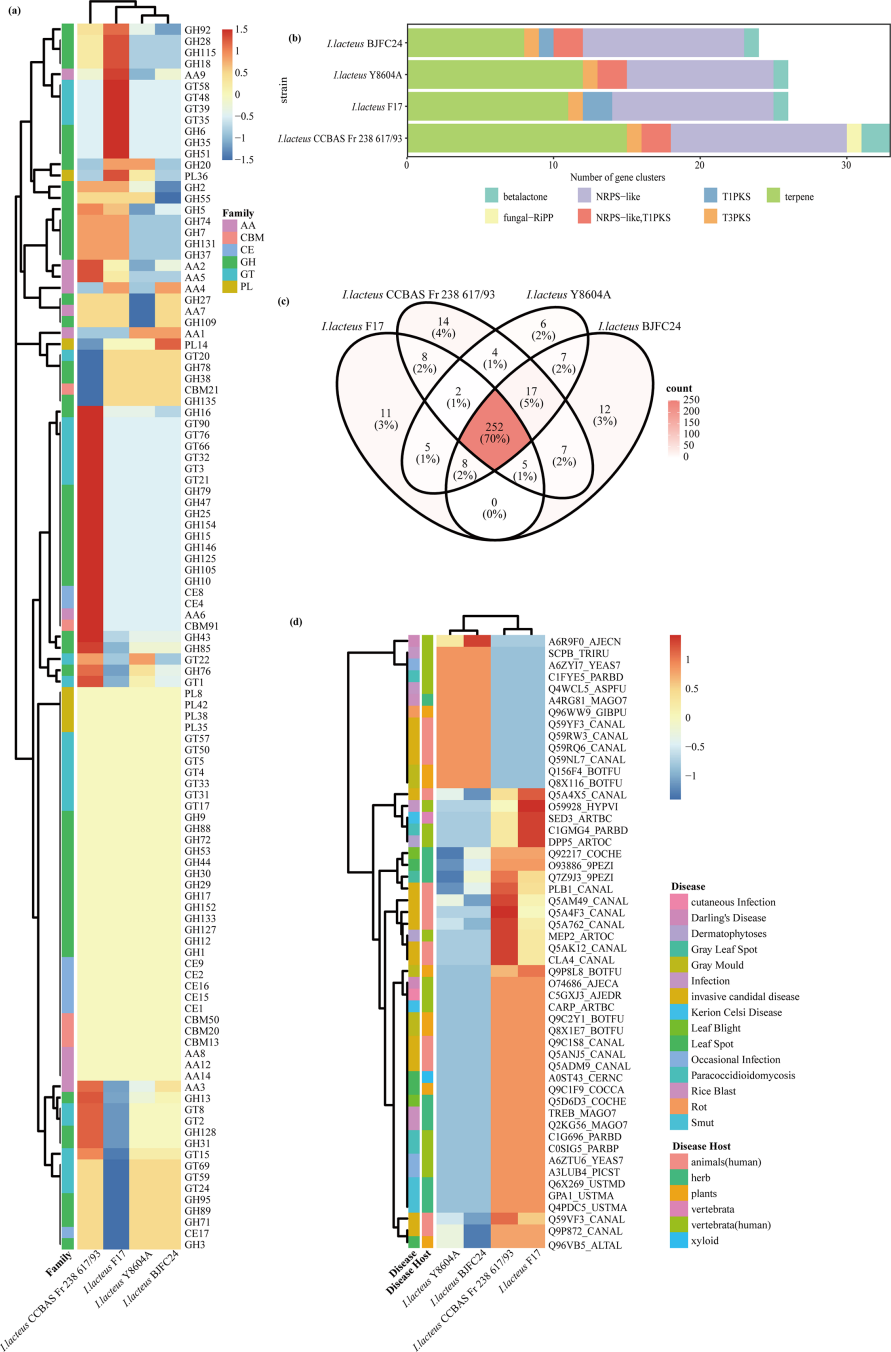
Comparative genomic features of *I. lacteus* strains. (**a**) CAZy annotation of *I. lacteus*. (**b**) Predicted secondary metabolism gene clusters in the genome of *I. lacteus* strains. (**c**) Venn diagram of DFVF-annotated human disease-related virulence factor genes across four *I. lacteus* isolates. (**d**) The DFVF annotations of the strains were mapped to the PHI database to compare the distribution of predicted virulence-associated genes.

Previous studies have shown that the ability of micro-organisms to degrade and utilize sugars is closely linked to their adaptation to different habitats [48]. To explore this adaptation potential in *I. lacteus*, we conducted a comparative analysis of CAZymes. Environmental strains of *I. lacteus* also possessed a higher number of genes encoding 12 CAZyme families compared to human-derived strains (Fig. 5a and Table S7). These included enzymes such as AA2 (MnP) and AA5 (oxidase with oxygen as an acceptor) which were involved in the degradation of lignin and other components of the plant cell wall [49], GH37 (trehalase) which regulated the levels of trehalose [50] and several GHs [e.g. GH115 (xyloglucan *α*-1,2-glucuronidase), GH131 and GH18] which contributed to the degradation of plant cell walls. Conversely, human-derived strains showed a slightly higher number of genes annotated to AA1 (laccase), possibly aiding in colonization and infection processes, similar to its role in *C. neoformans* dissemination in lungs [51]. These comparative genomics trends align with the genomic features of BJFC24 itself. In the CAZyme-related gene annotations of BJFC24, most are concentrated in the AA and GH families, indicating that despite its origin from the human body, it retains a broad metabolic capacity. This retention may support both environmental adaptability and opportunistic colonization of the host.

To explore the potential role of secondary metabolism in environmental adaptation, comparative genomic analysis was performed on secondary metabolism genes across four *I. lacteus* strains from different sources (Fig. 5b, Table 3) using antiSMASH software. The environmental strain CCBAS Fr 238 617/93 exhibited the highest number of annotated secondary metabolic functional gene clusters, whilst strain BJFC24, isolated from the reproductive tract, had the fewest terpene-annotated gene clusters amongst four strains (Fig. 5a and Table S8). Furthermore, *I. lacteus* CCBAS Fr 238 617/93 uniquely possessed a type of fungal ribosomally synthesized and post-translationally modified peptide (fungal-RiPP) cluster, along with a higher number of gene clusters annotated to betalactone compared to the other three strains. Fungal-RiPPs serve a defensive function [52], whilst betalactones exhibit potent antimicrobial activity [53,54]. However, the human-derived strains exist in relatively stable colonization environments that likely reduce the selective pressure for maintaining these gene clusters [6]. Notably, the annotation of BJFC24’s secondary metabolite gene clusters revealed that the NRPS-like-associated gene clusters were the most abundant, a feature not observed in the other three strains, which suggests that *I. lacteus* colonizing the female reproductive tract has undergone a unique ecological adaptation.

##### Comparative genomic analysis of potential pathogenicity genes

To evaluate the virulence factors in *I. lacteus* that are potentially associated with human diseases, we analysed their presence and absence across four isolates from different sources (Fig. 5c and Table S9). The Venn diagram shows that the majority (70%) of these virulence factors are shared amongst all four isolates, indicating that they belong to the core genes of *I. lacteus*. Furthermore, several (7%) virulence factors were found exclusively in the human-derived strains.

To explore the potential pathogenicity and host-pathogen interactions of *I. lacteus* strains, we analysed gene annotations from PHI and the DFVF databases. When comparing environmental and human-derived strains, the latter exhibited unique patterns of virulence-related genes. Specifically, mapping DFVF database-predicted genes to the PHI database identified 13 virulence factors with a higher number of predicted genes in human-derived strains, 9 of which were associated with human diseases, such as Darling’s disease, invasive candidal disease, infection, paracoccidioidomycosis and occasional infection (Fig. 5d and Table S10). In addition, seven of these virulence factors were uniquely annotated in human-derived strains.

In summary, although most DFVF-predicted genes associated with human diseases are conserved within the *I. lacteus* species, the seven unique virulence factors and their associations with human diseases in *I. lacteus* strains isolated from human sources suggest their potential risk of causing infections, particularly in immunocompromised individuals. However, further studies are needed to determine the functional roles of these genes in pathogenicity.

## Discussion

This study reports the first isolation of *I. lacteus* from the human cervix uteri. Known for colonizing dead tree trunks and its lignin degradation capabilities [3,6, 7], *I. lacteus* has also been identified as a potential pathogen in immunocompromised individuals [12,14]. Therefore, the isolation of BJFC24 highlights the need to investigate its adaptability (opportunistic colonization), potential pathogenicity and interactions with the human immune system.

### Adaptation of *I. lacteus* BJFC24 to the human reproductive tract

In the study, we collected and cultured cervical swabs under strict aseptic conditions to prevent contamination. The only other micro-organisms isolated alongside *I. lacteus* BJFC24 were typical members of the female reproductive tract (e.g. *Lactobacillus* and *Candida*) [55,57]. Unlike its spore-producing counterparts in natural environments [3,7], BJFC24 appeared as a nonsporulating white mould, aligning with observations of *I. lacteus* isolates from *C. hawkesii* Gray and clinical isolates of filamentous basidiomycetes [9,12,14, 58], implying that its presence was not likely due to laboratory contamination.

To further assess the genomic similarity of BJFC24, we conducted ANI analyses, which did not reveal a closer relationship between BJFC24 and any of the other three strains. Through a telephone follow-up, we learnt that the patient’s workplace is filled with numerous plants. Based on this information, we speculated that frequent exposure to plants could have facilitated the transfer of *I. lacteus* to the human reproductive tract. Definitely, many other potential routes of entry exist. Further research will be necessary to explore these possibilities. Genomic variation analyses indicated that environmental conditions influence intraspecific variation in *I. lacteus*. As shown in Fig. 3, InDel and SNP variations were primarily concentrated in upstream and downstream regions, which are generally noncoding regions that harbour regulatory elements such as promoters and enhancers involved in regulating gene expression [61]. Variations within exon regions which are part of coding areas were less frequent. Nevertheless, these exonic variations may alter protein expression [62] and potentially affect the strain’s adaptability to diverse ecological niches. Basic functional genomic annotation of BJFC24 revealed its ability to sense host signalling molecules, modify protein, manage substance transport and metabolism and interact with the host immune system, all of which contribute to its growth and adaptation in this niche. Furthermore, in the antiSMASH annotation of BJFC24, NRPS-like biosynthetic gene clusters were the most prevalent amongst all predicted cluster types, a prominent feature not observed in the other three strains. It indicates that the increased number of NRPS-like gene clusters in BJFC24 may contribute to the production of nonribosomal peptides (NRPs) with distinct bioactivities, including immunomodulatory or microbiota-modulating functions [47,63], which potentially may support its adaptation to the microenvironment of the female reproductive tract and facilitate its opportunistic colonization.

### Immune evasion and asymptomatic colonization

Compared to *I. lacteus* environmental strains, BJFC24 retained certain CAZymes linked to plant-associated traits and exhibited more laccase-related genes. Laccases, known for their role in the pathogenicity of *C. neoformans* [51], may suppress host immune responses [64] and protect against environmental stressors [65], which might endow *I. lacteus* BJFC24 with the ability to colonize human cervix uteri. The unique pathways for flavonol and peptidoglycan biosynthesis can modulate immunity and inhibit inflammation [66,67], with peptidoglycan also having anti-inflammatory effects [68]. These features underscore the specific adaptation of BJFC24 to the reproductive tract and its potential to interact with the human immune system to facilitate stable colonization. However, further research is required to clarify the distribution and roles of these pathways in the human body and their impacts on BJFC24’s colonization in the reproductive tract.

Additionally, BJFC24 also harboured genes for NRPS, which are involved in synthesizing pharmacologically active NRPs [47,69]. These peptides, known for their immunosuppressive effects [47,63], could help explain the absence of infection symptoms in the host. Previous studies have reported that *I. lacteus* can persist asymptomatically in immunocompromised hosts due to low virulence and immune factors [14]. In this case, immune dysregulation due to COVID-19 [70] likely enabled colonization. Similarly, filamentous fungi have been reported to coexist with the vaginal microbiota without causing disease [57,71].

### Latent pathogenic potential of *I. lacteus* BJFC24

Whilst the colonization of BJFC24 in this case remained asymptomatic, its genomic profile suggests latent pathogenic potential. PHI and DFVF analyses indicate low virulence but potential pathogenicity under certain conditions. As shown in Table 4, a comparative analysis with 17 closely related species revealed the highest homology with *C. albicans*, a common commensal fungus in the reproductive tract that occasionally causes opportunistic infections [57,72]. Other homologous species, such as *C. neoformans* [73,74], *N. fumigata* (formerly *Aspergillus fumigatus*) [75], *C. glabrata* [76] and *C. gattii* [77], are associated with severe infections, particularly in immunocompromised patients. This suggests that BJFC24 may possess opportunistic infection potential similar to that of the aforementioned fungi during its colonization in the human body. Therefore, based on the current study, we propose that BJFC24 isolated from the female cervix should be regarded as a colonizing fungus with latent pathogenic potential, whilst its pathogenic microbial properties remain to be further validated. In the future, additional experimental studies, such as virulence assays and host immune response analyses, are necessary to confirm any pathogenic potential.

### Comparative genomic insights across environmental and human-derived isolates

Beyond the strain-specific features of BJFC24, we also examined broader trends across all four isolates. Compared to human-derived strains, environmental *I. lacteus* isolates exhibited a broader functional gene repertoire across multiple analyses (KOG, GO and KEGG), especially in pathways related to signal transduction, metabolism and stress response. For instance, the MAPK signalling pathway plays a central role in environmental strains' responses to various environmental stresses [78], reflecting their adaptation to more variable and challenging natural environments. Notably, environmental strains of *I. lacteus* harboured an increased number of genes encoding CAZymes related to plant cell wall degradation, reflecting their capacity to utilize diverse environmental carbon sources [8]. In particular, CCBAS Fr 238 617/93 exhibited a higher count of gene clusters associated with fungal RiPPs, which serve defensive functions [52], and *β*-lactones, known for their antimicrobial activity [53,54]. These features likely enhance the strain’s ability to inhibit competing micro-organisms, enabling it to occupy ecological niches more effectively, defend against microbial threats and survive under fluctuating environmental conditions.

Previous studies on *I. lacteus* have primarily focused on its lignin degradation capabilities [7]. For instance, the F17 strain exhibits a significant enrichment of genes related to MnP. Research on the CCBAS Fr 238 617/93 strain has provided genomic insights into evolutionary adaptations associated with wood decay [6]. These environmental strains demonstrate robust secretion of laccase and MnP enzymes [5,79]. This study also observed this phenomenon, with AA2 (MnP) being more abundantly annotated in environmental strains (see Table S7). Human-derived strains, however, contain a higher number of genes related to AA1 (laccase), indicating that they retain key lignin degradation features characteristic of environmental *I. lacteus* strains. Additionally, F17 possesses a rich array of CAZymes involved in plant cell wall degradation [7], aligning with our current findings. In this study, genes such as GH5 (endo-beta-1,4-glucanase/cellulase) were found to be more abundantly annotated in environmental strains (see Table S7).

Compared to environmental strains, human-derived strains exhibited higher gene counts in GO annotations related to COPII receptor activity and kinetochore, suggesting that they may adapt to the human host environment by enhancing intracellular vesicle assembly and transport capabilities [80], as well as enhancing cell division capacity [81]. Additionally, KEGG analysis revealed an increased number of genes associated with taurine and hypotaurine and d-amino acid metabolism in human-derived strains. These amino acids, present in the female reproductive tract and gut [82,85], may aid in modulating immune responses and maintaining microbial balance, reflecting the strains' adaptation to the host environment [83,86].

Notably, core genes linked to human pathogenicity are ubiquitous across *I. lacteus* strains from any ecological niche. Strain-specific genes account for only a small proportion, and amongst these, no strain-specific virulence patterns were identified. This indicates that these isolates may have the genetic potential to cause opportunistic infections in immunocompromised hosts, with disease manifestation depending on host factors such as immune status. The human-specific virulence factors may contribute to opportunistic colonization of the reproductive tract or gut (Table S10).

Overall, comparative genomic analyses revealed the dual ecological adaptability of *I. lacteus* across environmental and human-associated niches and serve as a reference for its evolutionary trajectory between these distinct habitats. Based on our genomic annotation of BJFC24, the strain retains its lignin-degrading capabilities whilst also exhibiting traits that facilitate adaptation to the human reproductive tract environment. This dual functionality explains how *I. lacteus* can both persist in wood and colonize the human reproductive tract.

## Conclusion

In summary, this study reports the first isolation of *I. lacteus* from the female reproductive tract and provides a detailed morphological characterization of this strain. Notably, this filamentous fungus did not produce spores and did not induce infection symptoms during its colonization of the human reproductive tract, despite its ability to survive at 37 °C, the human body temperature. Genome sequencing revealed mechanisms underlying its adaptation to this niche, which could be related to its interactions with the host immune system, potentially contributing to the absence of clinical symptoms. However, the genome also indicates potential risks for human infection, warranting further investigation into the clinical relevance of *I. lacteus* in human health.

## Supplementary material

10.1099/mgen.0.001416 Uncited Supplementary Material 1.

10.1099/mgen.0.001416 Uncited Supplementary Material 2.

## Abbreviations

AAs: auxiliary activity enzymes
ANI: average nucleotide identity
BI: Bayesian inference
CAZymes: carbohydrate-active enzymes
CB: cotton blue
CBM: carbohydrate-binding module
COPII: coat protein complex II
COVID-19: coronavirus disease 2019
DFVF: Database of Fungal Virulence Factors
DNBs: DNA nanoballs
fungal-RiPP: fungal ribosomally synthesized and post-translationally modified peptide
GHs: glycoside hydrolases
GO: Gene Ontology
InDels: insertions/deletions
ITS: internal transcribed spacer
KEGG: Kyoto Encyclopedia of Genes and Genomes
KOG: EuKaryotic Orthologous Groups
KOH: potassium hydroxide
MAPK: mitogen-activated protein kinases
ML: maximum likelihood
MnP: manganese peroxidase
NMDC: National Microbiology Data Center
NRPs: nonribosomal peptides
NRPS-like: non-ribosomal peptide synthetase-like
PDA: potato dextrose agar
PHI: Pathogen-Host Interaction
PP: posterior probabilities
SEM: scanning electron microscopy
T1PKS: type I polyketide synthase
