# Supplementary material for: Whole-genome sequencing and comparative genomic analysis of Irpex lacteus isolated from the female reproductive tract
Source: Microb Genom. 2025 Jun 3;11(6):001416. doi: 10.1099/mgen.0.001416 (PMC12134385; doi:10.1099/mgen.0.001416)
Supplement: Uncited Supplementary Material 1. [file mgen-11-01416-s001.pdf]

Supplementary Materials for

Title: Whole Genome Sequencing and Comparative Genomic Analysis of *Irpex lacteus* Isolated from the Female Reproductive Tract in China

Authors: Yixuan Wang[1,3†], Shujuan Zhang[2,3†], Shiling Han[4,5], Xiaomeng Ge[6], Shenghan Gao[3], Qianhui Zhu[3], Yadong Liu[3,5], Songnian Hu[3,5], Ziwen Jiang[7], Yinmei Dai[7], Lei Cai[4,5], Yu Vincent Fu[3,8,9*]

Corresponding author:fuyu@im.ac.cn

Supplemental Figures

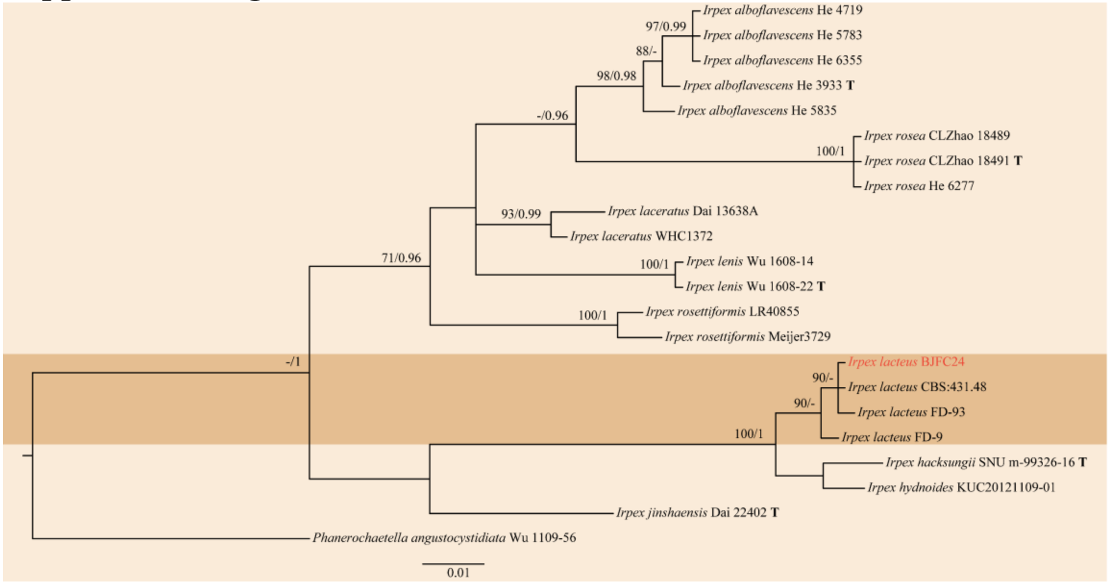

Fig. S1. Phylogenetic tree inferred based on the ITS regions of *Irpex* species, with *Phanerochaetella angustocystidiata* Wu 1109-56 as the outgroup. Strains isolated in this study are marked in red. RAxML support values (ML-BS ≥ 70%) and Bayesian posterior probabilities (BI-PP ≥ 0.90) are shown. Type strains (ex-type) are represented in bold with T.

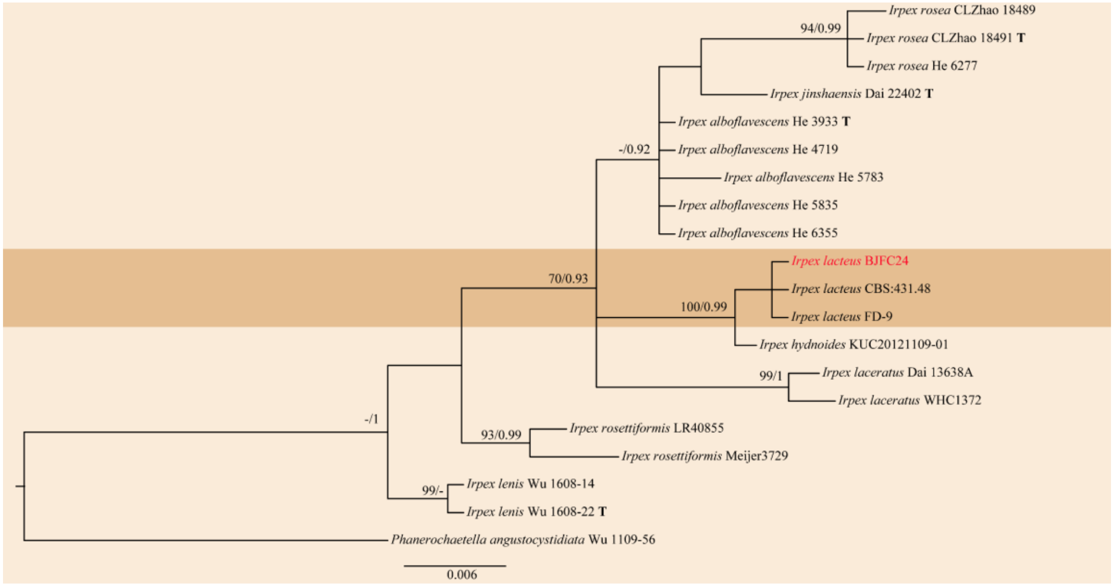

Fig. S2. Phylogenetic tree inferred based on the LSU ribosomal RNA regions of *Irpex* species, with *Phanerochaetella angustocystidiata* Wu 1109-56 as the outgroup. Strains isolated in this study are marked in red. They are in the same clade as *I. lacteus* CBS:431.48 and *I. lacteus* FD-9. RAxML support values (ML-BS ⩾ 70%) and Bayesian posterior probabilities (BI-PP ⩾ 0.90) are shown. Type strains (ex-type) are represented in bold with T.

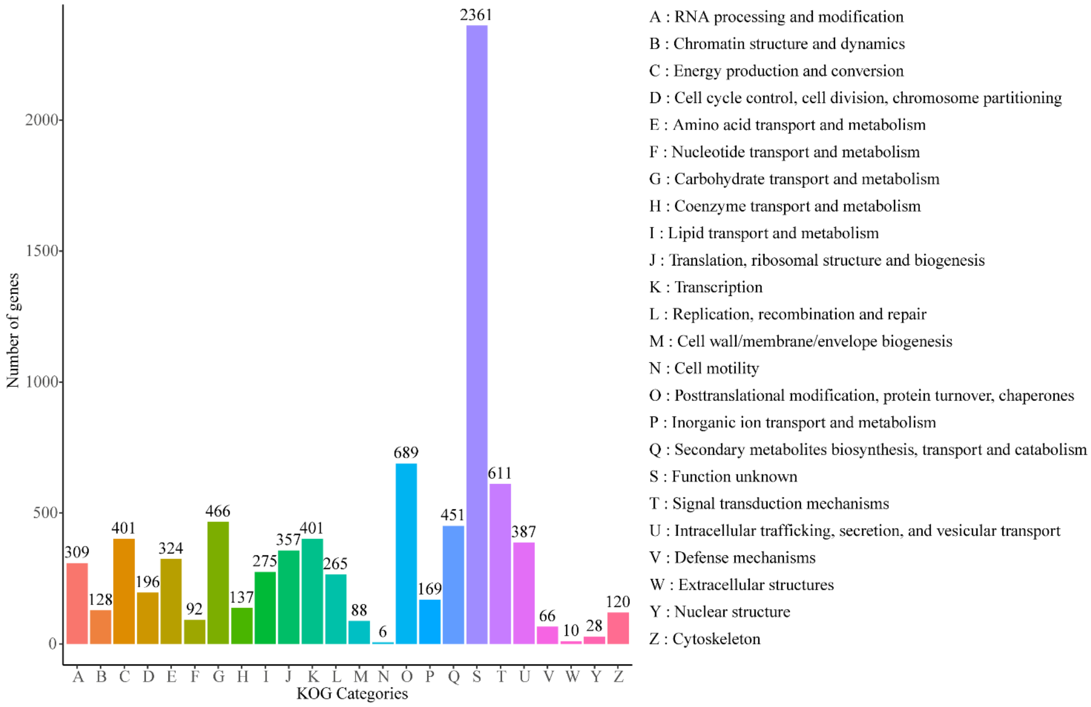

Fig. S3. KOG classification of predicted genes in *Irpex lactues* BJFC24.

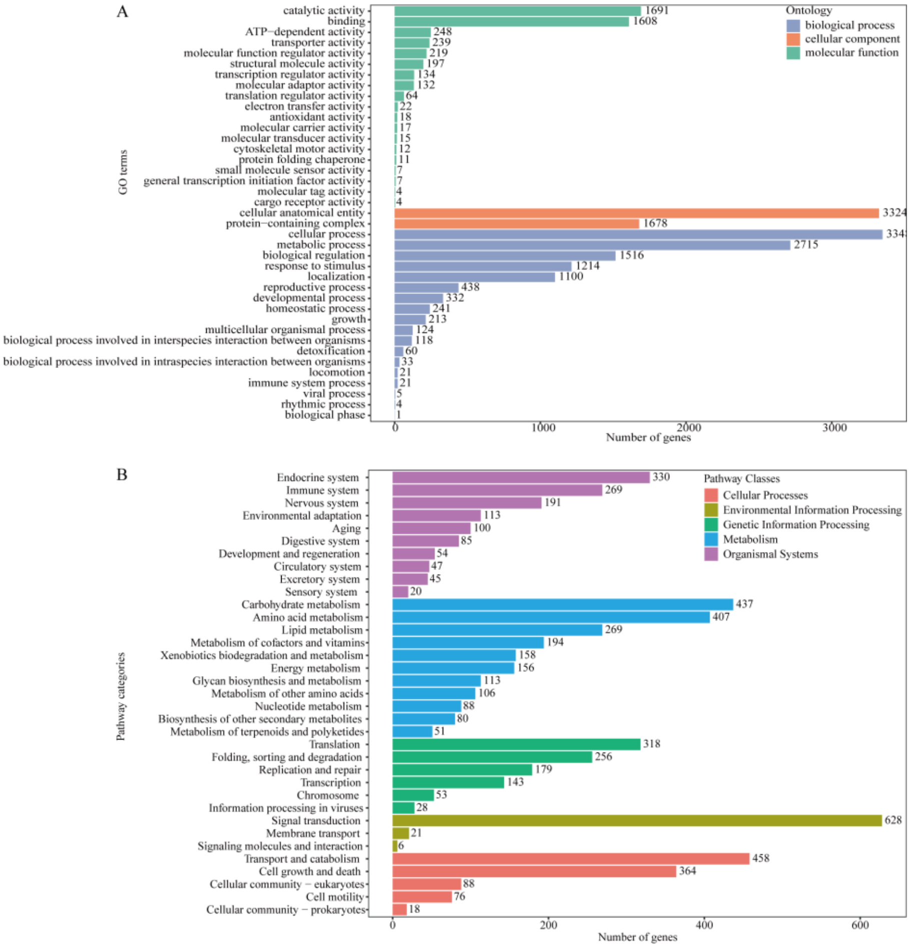

Fig.S4. GO(A) and KEGG(B) classification of predicted genes in *Irpex lactues* BJFC24.

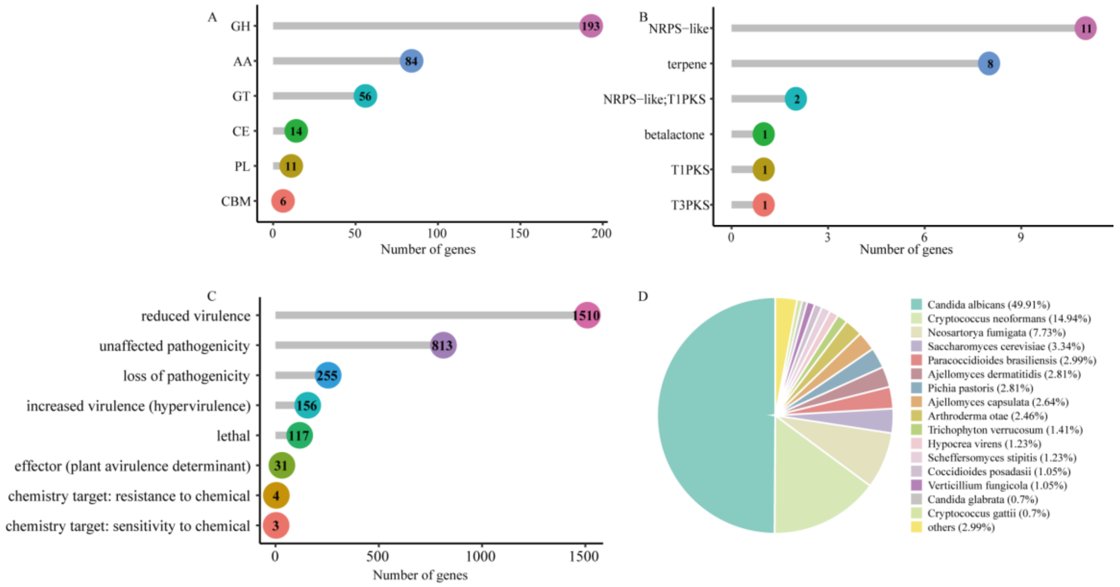

Fig.S5. Protein annotation in *Irpex lactues* BJFC24. (A) The distribution of CAZymes categories. GH: glycoside hydrolases, AA: auxiliary activities, GT: glycosyltransferases, CE: carbohydrate esterases, PL: polysaccharide lyases, CBM: carbohydrate-binding modules. (B) Predicted gene clusters from antiSMASH. (C) The distribution of pathogen-host interaction (PHI) genes. (D) The homologous species distribution based on the database of fungal virulence factors (DFVF).

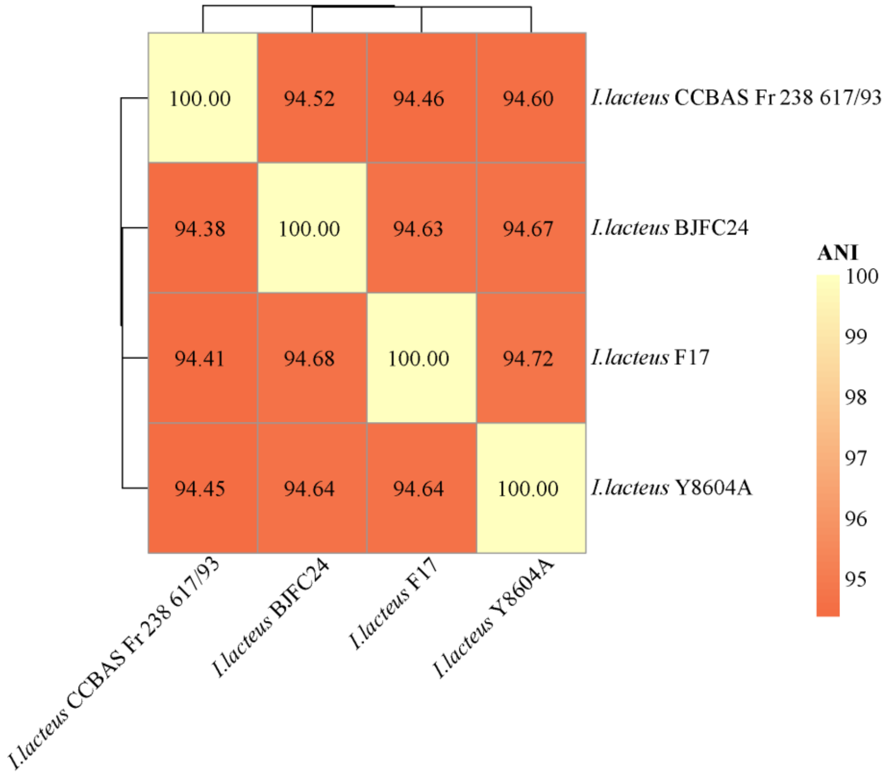

Fig.S6. ANI-based genomic relatedness among *I. lacteus* strains
